# Global and Local Architecture of the Mammalian microRNA–Transcription Factor Regulatory Network

**DOI:** 10.1371/journal.pcbi.0030131

**Published:** 2007-07-13

**Authors:** Reut Shalgi, Daniel Lieber, Moshe Oren, Yitzhak Pilpel

**Affiliations:** 1 Department of Molecular Genetics, Weizmann Institute of Science, Rehovot, Israel; 2 Department of Molecular Cell Biology, Weizmann Institute of Science, Rehovot, Israel; Washington University, United States of America

## Abstract

microRNAs (miRs) are small RNAs that regulate gene expression at the posttranscriptional level. It is anticipated that, in combination with transcription factors (TFs), they span a regulatory network that controls thousands of mammalian genes. Here we set out to uncover local and global architectural features of the mammalian miR regulatory network. Using evolutionarily conserved potential binding sites of miRs in human targets, and conserved binding sites of TFs in promoters, we uncovered two regulation networks. The first depicts combinatorial interactions between pairs of miRs with many shared targets. The network reveals several levels of hierarchy, whereby a few miRs interact with many other lowly connected miR partners. We revealed hundreds of “target hubs” genes, each potentially subject to massive regulation by dozens of miRs. Interestingly, many of these target hub genes are transcription regulators and they are often related to various developmental processes. The second network consists of miR–TF pairs that coregulate large sets of common targets. We discovered that the network consists of several recurring motifs. Most notably, in a significant fraction of the miR–TF coregulators the TF appears to regulate the miR, or to be regulated by the miR, forming a diversity of feed-forward loops. Together these findings provide new insights on the architecture of the combined transcriptional–post transcriptional regulatory network.

## Author Summary

It is becoming increasingly appreciated that a new type of gene which does not code for proteins, the regulatory RNAs, constitutes a considerable portion of mammalian genomes, and these genes serve as key players in the regulatory network of living cells. Among these regulatory RNAs are the microRNAs (miRs), small RNAs that mediate posttranscriptional gene silencing through inhibition of protein production or degradation of mRNAs. So far little is known about the extent of regulation by miRs, and their potential cooperation with other regulatory layers in the network. We investigated the potential crosstalk between the miR-mediated posttranscription layer, and the transcriptional regulation layer, whose dominant players, the transcription factors (TFs), regulate the production of protein-coding mRNAs. We found that the extent of miR regulation varies extensively among different genes, some of which, especially those who serve as regulators themselves, are subject to enhanced miR silencing. Further, we identified thousands of genes that are potentially subjected to coordinated regulation by multiple miRs and by specific combinations of TFs and miRs. The regulatory network, comprising transcriptional and posttranscriptional regulation, manifests several recurring architectures, one of which consists of a TF and a miR that together regulate a large set of common genes, and that also appear to regulate one another. Altogether this work provides new insights into the logic and evolution of a new regulatory layer of the mammalian genome, and its effect on other regulatory networks in the cell.

## Introduction

microRNAs (miRs) are short RNAs that post transcriptionally regulate messenger RNAs. Two main mechanisms for such effects are degradation of the target mRNA, and inhibition of its translation [1]. In recent years considerable progress within multiple genomes was obtained in the experimental identification of genes encoding for miRs [2–4], and in tools for the identification of target genes of miRs, based on miR sequences and the sequence of the targets' 3′ untranslated regions (UTRs) [5–11]. Compared with the regulation of transcription, the study of the regulatory networks spanned by miRs is only at its beginning. When it comes to transcriptional regulation, a lot is known about the main players and the interactions between them. Transcription factors (TFs) are well-characterized [12], and promoter binding motifs are available in a diversity of species [13]. The combinatorial interactions between TFs have been explored [14,15] as well as the global level properties of the transcription regulatory network [16]. In addition, the local structures of the network have been intensively investigated. It was found in several species that the transcription regulatory network may be decomposed into elementary building blocks, or network motifs, that recur in the network more than expected by chance, and that these motifs likely perform local “computations,” such as the detection of signal persistency or the coordinated gradual activation of a set of genes [17–20].

When it comes to posttranscriptional regulation, and in particular to the miR world, most of the parallel knowledge is lacking. While we do know about many miRs in multiple genomes [1], their targets are predicted with relatively limited accuracy [21]. Even more obvious is the lack of knowledge about the structure of the miR regulatory network, and about the potential interface between this network and the transcriptional one. In similarity to TFs, miRs are expected to work in combinations on their target genes [7]. The target specificity-determining site of the miRs is often short (seven to eight nucleotides [9]), hence some genes that contain a match to a single miR in their 3′ UTRs may represent false positive assignments. Thus, combinatorial interactions among the miRs are probably necessary to specify more precisely the set of affected targets of each miR. As in the realm of transcription regulators [14]*,* combinatorics may also have the advantage of allowing multiple sources of information, each represented by a single miR, to be integrated into the regulation of individual transcripts.

Since TFs regulate mRNA production, and miRs regulate transcript stability and its translation, an attractive possibility is that miRs and TFs cooperate in regulating shared target genes. This possibility is appealing since a gene that is regulated through multiple mechanisms may be tuned at a level of precision that is higher than what may be obtained by either mechanism alone. In addition, as with any other regulatory agent in cells, the question “what regulates the regulator” is of prime importance, as it may allow the exposure of multiple levels of hierarchies and their design within a control network. It is thus crucial to understand whether TFs and miRs collaborate in gene regulation, and also to characterize regulatory interactions that miRs and TFs may exert on each other. In similarity to the transcription network, local network motifs might exist which may also consist of miRs. One attractive role for such motifs has been suggested in a developmental context—to canalize “noise” in gene expression [22]. However, actual realization of such motifs remains to be explored.

Here we report extensive combinatorial interactions among miRs and between miRs and TFs. We found hundreds of miRs target hubs—genes regulated by dozens of miRs—which are involved in a diversity of developmental processes and in transcription regulation. The miR–TF regulatory network features several motifs in which TF and miR partners that are suggested to regulate multiple target genes often exert regulation on one another.

## Results

### Connectivity Distributions in the miR–Gene Network

We used two datasets of miRs and their predicted target genes: TargetScan [8,9] and PicTar [7]. The miRs used in this analysis are characterized by being evolutionarily conserved, and, in addition, their targets were defined based on conservation in orthologous genes in four species (human, mouse, rat, and dog). This evolutionary conservation criterion was assumed to constitute a good filter for false positive assignments of miRs to genes [9,23]. Yet, it must be emphasized that the accuracy of such assignments is still limited [21] (see “noise tolerance analysis” in Materials and Methods). Altogether we analyzed 8,672 and 9,152 human (RefSeq) genes in the TargetScan and PicTar datasets, respectively, that have at least one predicted miR binding site in their 3′ UTR, and a total of 138 miRs and 178 miRs in the respective datasets.

**Figure 1:**
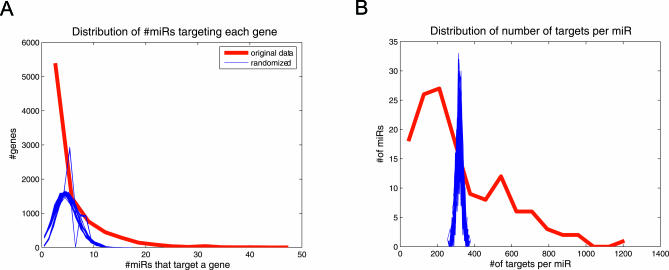
miRs and Target Genes in the TargetScan Dataset (A) Distribution of the number of different miRs regulating each target gene in the TargetScan dataset. The thick red line represents the distribution in the original datasets, while each of the thin blue lines represents the distribution in one of the column-randomized matrices. The matrix contains only genes with at least one predicted site in their 3′ UTR. In each randomization, we shuffled the assignment of miRs to their targets, keeping constant the number of targets per miR. (B) Distribution of number of targets per miR in the TargetScan dataset. In the thick red line we depicted the original distribution, while each blue thin line represents the distribution in one of the 100 row-randomized matrices, which preserve the distribution of number of miRs targeting each gene.

We constructed a matrix whose rows are genes and columns are miRs, in which the ij-th element is “1” if gene i contains a predicted binding site for miR j in its 3′ UTR, and “0” otherwise. We created one such matrix for each of the two miR target prediction datasets. For the sake of clarity, from here on we will say interchangeably that “a miR targets a gene” or that “a gene contains in its 3′ UTR a predicted binding site for a miR.” We first characterized the matrix by the distribution of degree connectivity of each gene and of each miR. Figure 1A shows the distribution of the number of miRs assigned per gene, while Figure 1B shows the distribution of number of genes assigned to each miR. We compared each distribution with a set of distributions, each derived by randomization of the original matrix according to two alternative null models. Along with the distribution of number of miRs per gene (Figure 1A), we also plotted 100 distributions obtained after randomizing each of the columns in the matrix. In this randomization we preserved the number of genes per miR, yet assigned genes at random to each miR. The distributions obtained after the randomization differed markedly from the original distribution, both in terms of width and shape. While in the randomized distributions genes rarely have more than ten different miRs in their 3′ UTR, in the original distribution there are hundreds of genes subjected to extensive predicted miR regulation. In Figure 1B we also show the distribution of number of genes per miR. Along with it is shown a set of distributions obtained by randomizing each of the rows in the matrix, namely by randomly assigning miRs to each gene, preserving the real number of miRs predicted to target each gene, as in the original matrix. Here, too, the randomized distributions differed from the original one both in shape and width; the original data contains multiple miRs which appear to target more than 400 genes, significantly higher than the number that would be obtained by merely preserving the statistics of number of miR sites in genes UTRs. These observations lead us to highlight some special properties that seem to be unique to the miR regulatory network.

### Target Hubs—Genes with Extensive miR Regulation

The distribution of number of miRs regulating each target gene (Figure 1A) has a long right tail in contrast to the distributions in the randomized matrices that looked Gaussian (as befits a sum of independent random variables). We thus focused on the genes in that tail of the distribution (which are targeted by more than 15 miRs and 20 miRs in the TargetScan and PicTar datasets, respectively; see Materials and Methods for further details and cutoff justification). We named these genes target hubs following a recent definition of genes regulated by multiple TFs in yeast [24]. There are 470 such genes in the TargetScan dataset. We made similar observations with the PicTar dataset and identified 834 target hubs (see Figure S1)—the set of target hubs based on the TargetScan dataset has an 81% overlap with the target hubs defined by PicTar dataset.

Inspecting the target hubs genes' annotations (using Gene Ontology, GO), we found that they are highly enriched for developmental processes, specifically for muscle development and nervous system development, as well as for TFs and transcription regulators (see Table 1 for enrichment statistics). Among the transcription regulators in the set of target hubs are included RUNX1, E2F-3, N-MYC, and SP3. Another very intriguing fact is that the *Ago1* gene, one of the key components of the human RISC (RNAi induced silencing complex), is also a target hub, as in the dataset it appears to be potentially regulated by multiple miRs.

**Table 1:**
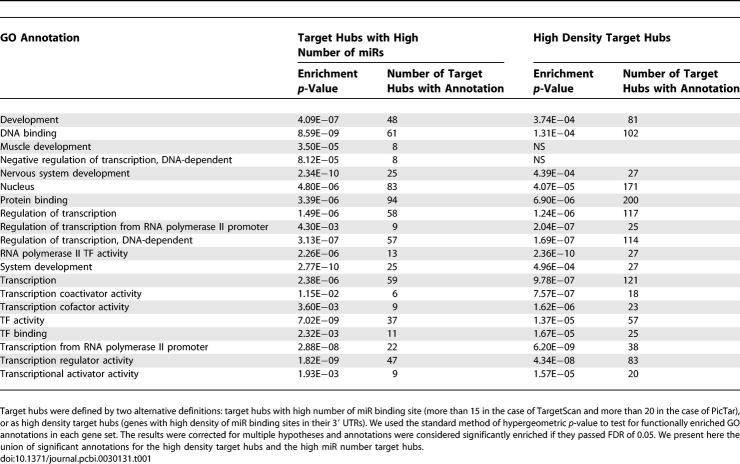
TargetScan Target Hubs GO Functional Enrichment

**Figure 2:**
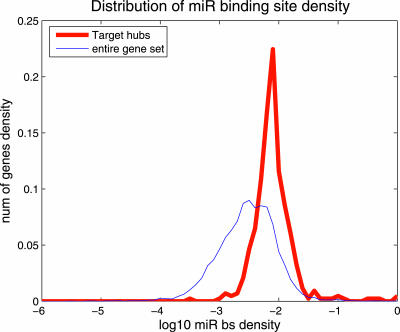
Distribution of the density of miRs in the 3′ UTRs of target hubs (thick red line) and all the genes (thin blue line) in the TargetScan dataset (all genes included in this figures have at least one miR site predicted in their 3′ UTR). The log10 densities were binned into bins of 0.1, and relative frequencies were plotted. Same analysis for the PicTar dataset is in Figure S2.

We suspected, however, that the fact that target hubs host many miR binding sites may result from potentially longer 3′ UTRs [23]. Although we found that target hubs have a distribution of 3′ UTR lengths that is significantly longer than that of the rest of the genes in the current analysis (*p*-value = 4 × 10^−85^ and *p*-value = 3 × 10^−101^ for TargetScan and PicTar target hubs, respectively, using the Kolmogorov-Smirnov test), we still realized that many of them have relatively short 3′ UTRs (Figure S2A and S2B). To test whether the high number of miR binding sites in the target hubs is a simple reflection of their 3′ UTR lengths, we performed a randomization test, in which we sampled 100 times random gene sets from the entire dataset with the same or very similar length distributions as that of the target hubs (see Materials and Methods). We found that such gene sets always have a significantly lower average number of miR sites per gene compared with the target hubs (see Figure S3A). We further calculated the density of different miRs in the 3′ UTRs [23]. Density was defined as number of different miRs targeting a gene divided by 3′ UTR length. Remarkably, we found that the miR density in the target hubs is significantly higher than in the rest of the genes in the dataset (*p*-value = 2 × 10^−85^ and *p*-value = 6 × 10^−124^ for the TargetScan and PicTar target hubs, respectively, using the Kolmogorov-Smirnov test; the means are 2.84 and 1.80 times higher in the TargetScan and PicTar dataset means, respectively; see Figure 2 and Figure S2C for the entire distributions). We concluded that target hubs are rich in binding sites for different miRs to an extent that cannot be explained solely by their 3′ UTRs lengths.

Realizing that density of miR binding sites may be an important property by itself, we also used an alternative definition for target hubs—genes with particularly high density of miRs in their 3′ UTRs. We collected the genes in the top 85th percentile of the miR binding site density spectrum, then we performed a similar GO enrichment analysis to see whether particular functionalities were enriched among the genes with a high density of miR binding sites. Reassuringly, most of the functionalities that were enriched among the set of target hubs defined by number of differnet miRs were also significant in the set of high density target hubs (see Table 1). Moreover, we found that genes that were target hubs according to only one of the two definitions (i.e., genes that are not in the overlap of the two sets) were still significantly enriched for functionalities such as transcription regulator activity and development (unpublished data).

### A Combinatorial Network of miR Interactions

Combinatorial interactions are a fundamental property of the transcription networks [25]. It may be anticipated that, similarly to TFs, miRs may work in combinations. One way to predict pairs of coregulating miRs is to ask which pairs show a high rate of co-occurrence in the same target genes' 3′ UTRs. A common statistical test in the field, previously used in the context of promoter motifs and TF binding site [26–28], is the cumulative hypergeometric statistic. According to this model, given the rate of occurrence of each of the regulators alone, and the total number of genes in the analysis, a *p*-value is computed on the size of the set of genes that are shared between the two regulators. The main assumption of this model, that assignment of a gene to the first regulator is independent of the assignment to the second one, is likely fulfilled in the context of fixed-length promoters. Yet when it comes to 3′ UTRs of varying length, the assumption does not hold anymore. Some genes, e.g., those with long 3′ UTRs, have a higher chance to contain predicted binding sites for miRs, hence a *p*-value calculated based on the hypergeometric model may overestimate the significance of the co-occurrence rate.

We have thus devised an alternative, randomization-based test for identifying significantly co-occurring miR pairs. The model was designed such that it will capture the underlying distributions in Figure 1A and 1B, and test whether a given pair of miRs co-occurs at a higher rate, considering the above distributions as a background. For each pair of miRs, i and j, with their set of targets, Targets(*i*) and Targets(*j*), respectively, we calculated the “Meet/Min” score [29,30] defined in the present case as:

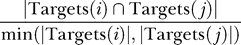
namely, the size of the set of genes that contain sites for the two miRs together, divided by the smaller of the two sets of targets (we filtered from the calculation for each *i*,*j* pair, 3′ UTRs in which the sites for *i* and *j* are physically overlapping to avoid overestimation of significance of miR pairs with an overlapping or similar seed, see Materials and Methods for details). Yet this score is not a statistic, i.e., it lacks an estimate of the probability to obtain such score (or better) by chance given an appropriate null model. Following previous works [20], we used a null model that preserves for each gene the number miRs assigned to it, and for each miR the number of genes assigned to it in the input data. We generated 1,000 randomized matrices according to this null model. In each such matrix we randomized the original matrix in 100,000 steps, using an edge-swapping algorithm [20]. For each such randomized matrix we computed again the Meet/Min score for all pairs of miRs. The co-occurrence *p*-value for a pair of miRs was computed according to the pair's Meet/Min score and the population of 1,000 Meet/Min scores obtained for that same pair in each of the 1,000 edge-swapped matrices. The *p*-value for the pair is defined as the fraction of the 1,000 randomized matrices in which the Meet/Min score of that pair is greater than or equal to the Meet/Min score of the pair in the original matrix.

In addition to calculating a score of co-occurrence, we also calculated, using the same formalism, a score that captures the tendency of every two miRs to avoid residing within shared 3′ UTRs. We will regard a pair of miRs that co-occur in the original matrix significantly less frequently than in the edge-swapped matrices as avoiding each other. Given the Meet/Min score of co-occurrence for a pair of miRs, and the Meet/Min scores obtained for that pair in the 1,000 edge-swapped matrices, we calculated the fraction of randomized scores that were lower than or equal to that obtained in the original matrix for that pair, as the avoidance *p*-value of a miR pair.

**Figure 3:**
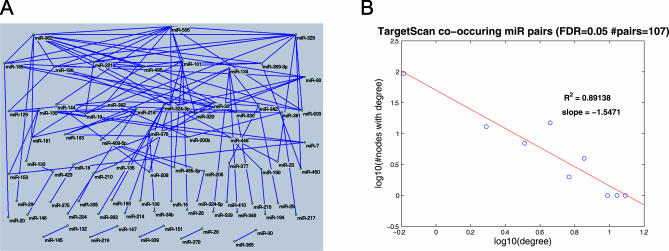
miR Co-Occurrence Network in the TargetScan Dataset (A) The TargetScan miR co-occurrence network, at FDR level of 0.05. A node represents a miR and an edge connects between pairs of miRs with significant rate of co-occurrence. The nodes in the figure are arranged from most highly connected on the top, to most lowly connected, on the bottom. For interactive viewing of the network, using Pajek (http://vlado.fmf.uni-lj.si/pub/networks/pajek/), see Datasets S1 and S2. (B) Degree distribution in the TargetScan miR combinatorial regulation network (co-occurring miR pairs that passed FDR of 0.05).

In both cases of co-occurrence and avoidance, we used the false discovery rate (FDR) to control for the testing of multiple hypotheses. In the case of co-occurring miR pairs, using a restrictive FDR threshold (*q*-value = 0.05), we obtained 107 pairs with a significant *p*-value in the TargetScan dataset, and 199 pairs in the PicTar dataset (interestingly, the ratio between the number of interactions in the two datasets (∼0.54) is very close to the ratio expected based on the square of relative number of miRs in each dataset (∼0.6)). We created a combinatorial network based on the significant co-occurring miR pairs. The top miR pairs are given in Table 2 and are also depicted in Figures 3A and S4A. The full list of significant pairs is provided in Tables S1 and S2. This combinatorial network consists of several levels of hierarchy. At the top (Figure 3A) are a handful of miRs that interact with a relatively large number of miR partners, while at the bottom are “end-nodes” with very few miR partners each. Examination of the degree distribution in the miR combinatorial network revealed a power law with a slope of about −1.5 and R^2^ = ∼0.89 in TargetScan and R^2^ = 0.94 in PicTar (Figures 3B and S4B), indicating that the network of coregulating miRs is scale-free (alternative FDR cutoffs also resulted in scale-free networks with R^2^ always bigger than 0.72). Interestingly, expression data of the miRs provides some support for the predicted regulatory interactions between them. We found that coexpressed miRs tended to have relatively high co-occurrence scores, and significant co-occurrence *p*-values, while miR pairs with negatively correlated expression tended to avoid residing in shared 3′ UTRs (see below).

**Table 2:**
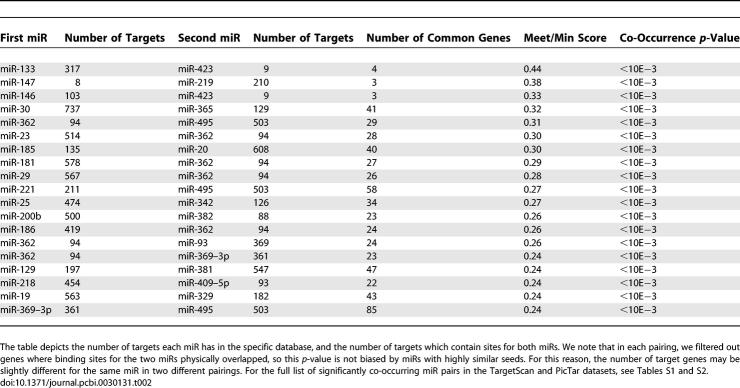
Top 20 Most Significant Pairs of Coregulating miRNAs in the TargetScan Network

### Coordinated Regulation of Target Genes by miRs and TFs

A potential regulatory design in the gene expression network is that genes belonging to the same regulon will be coregulated not only at the transcriptional level, but also posttranscriptionally [31]. One potential realization of this design may be that a particular miR and a particular TF would regulate common targets. A simple means to identify some of the cases of regulatory cooperation between a miR and a TF may be to find TF–miR pairs that co-occur in a large set of shared targets compared with the size expected by chance. Similar to the case of miRs sites in 3′ UTRs, we considered a TF to be present in a human gene's promoter only if its occurrence in the promoter is conserved in the promoters of orthologous genes from mouse and rat [32] (as taken from UCSC, see Materials and Methods). We then created a matrix whose rows are the genes and columns are TFs, with a “1” for the i-th gene and the j-th TF if the TF binding site (TFBS) occurs in the gene's promoter and “0” otherwise.

To identify pairs of TFs and miRs that cooperate in regulating shared target genes, we looked for TF–miR pairs with a high rate of co-occurrence in the promoters and 3′ UTRs of the regulated genes. We tested the co-occurrence in shared genes of each of the 409 position specific scoring matrices (PSSMs) representing TF binding sites in TRANSFAC [13] with each of the 138 and 178 miRs in the TargetScan and PicTar databases, respectively. A PSSM and a miR are said to co-occur in the same gene if the PSSM has a conserved binding site in the promoter of the gene and the miR has a conserved predicted site in the gene's 3′ UTR. We used two statistical models to calculate the significance of rate of TF–miR co-occurrence, and ultimately considered TF–miR pairs that were found to be significant according to both tests. First, a hypergeometric *p*-value was calculated based on the number of genes that contain a TFBS in their promoter, the number of genes that contain a miR site in their 3′ UTR, and the number of genes that contain both the TF and the miR sites (see Materials and Methods for details). We computed such *p*-values on all TF–miRs pairs and set a threshold on the *p*-values obtained to account for the multiplicity of hypotheses, using FDR. Using an FDR *q*-value of 0.3, we obtained 111 miR-TF pairs with significant *p*-values using the TargetScan dataset and 1,263 miR-TF pairs with significant *p*-values using the PicTar dataset (see Materials and Methods for number of pairs with more stringent *q*-values). Reassuringly, there is a high overlap between the TargetScan and PicTar networks (68.7% of the TargetScan miR–TF network pairs were also found to be significant pairs in the PicTar network). The hypergeometric *p*-value has the advantage of being an analytical model with essentially unlimited resolution. Also, unlike the above situation of miR co-occurring pairs, which exhibited inherent dependency between the two regulators, the present case of TF–miR interaction does not present such limitation (and is in fact identical to the classical cases in which hypergeometric model is used [33]). Nevertheless, we decided to also back up the hypergeometric-based predictions with a randomization test, very similar to the one presented above for the case of miR co-occurrence, that preserves the distribution of number of regulators of each gene, the number of targets of each TF, and the number of targets of each miR in the input datasets. We calculated the co-occurrence rates and *p*-values of all TF–miR pairs, and used FDR as above to account for the multiplicity of hypotheses (see Materials and Methods for details). Reassuringly, 93% and 72% of the hypergeometric-based TF–miR interactions from the TargetScan and PicTar datasets, respectively, were also supported by this alternative model. The rest of the analyses were based on TF–miR pairs that passed the two statistical tests using FDR; there were 104 pairs in the TargetScan dataset and 916 pairs in the PicTar dataset. For simplicity we term a TF and a miR that significantly co-occur as partners. Table 3 lists the top TF–miR partners. The full networks of TF–miR partners can be downloaded as Tables S3–S5, and interactively viewed in Datasets S3–S5.

**Table 3:**
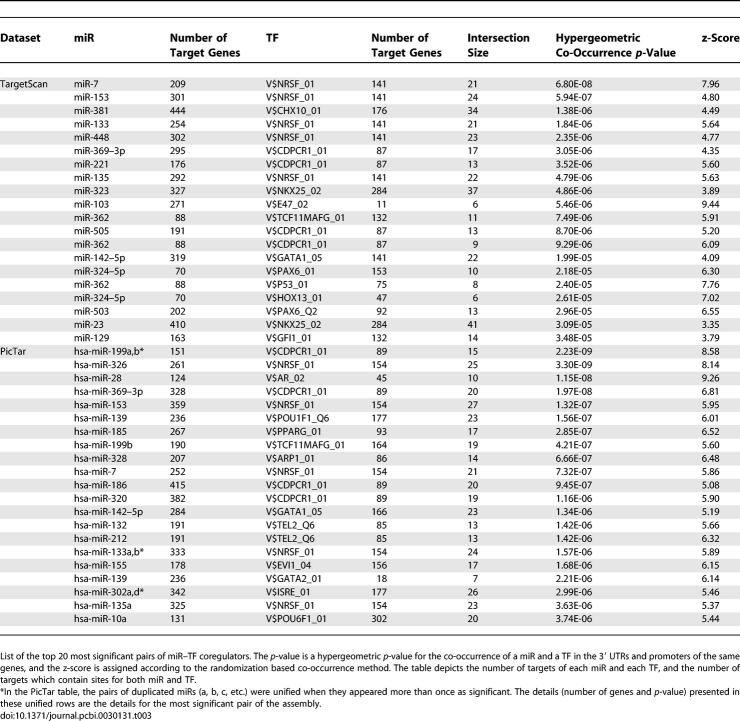
Top 20 Most Significant Pairs of Coregulating miRNAs and TFs in the TargetScan and PicTar Networks

### The Network of miR–TF Coregulation Reveals Recurring Local Architectures—Network Motifs

**Figure 4:**
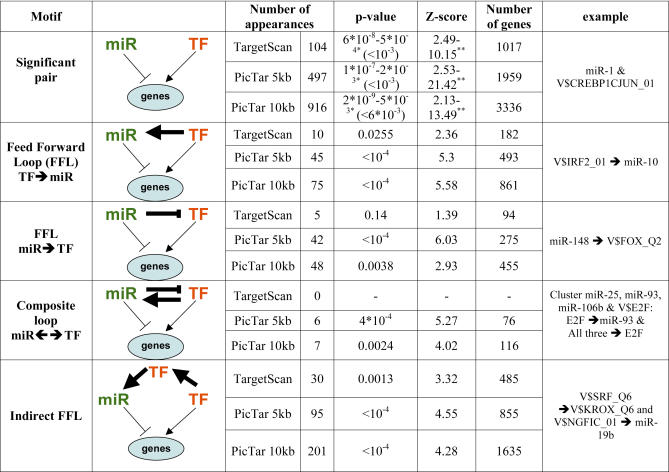
Network Designs in the miR–TF Coregulation Network The figure depicts the analyzed network motifs in the TargetScan and PicTar dataset, and with the use of TF binding sites in RefSeq genes promoters of 10 kb for both networks, and 5 kb for the PicTar network. The figure depicts, for each network motif, its architecture, the number of times it appears in each of the networks, the *p*-value and z-score for its over-representation in the network (as described in Materials and Methods), the total number of RefSeq genes that are regulated by this type of network design, and an example. *For the first design, the coregulating miR–TF pair, we state the range of hypergeometric *p*-values of pairs that passed FDR and are considered significant, and in brackets the FDR *p*-value of these pairs using the randomization co-occurrence test. **In addition, z-scores for significant pairs were calculated based on the co-occurrence edge-swapping randomization model (see Materials and Methods).

Recently it was suggested that in circuits composed of a miR and a TF, in which these two regulators target the same genes, the TF may also exert a regulatory effect on the miR with which it coregulates the target genes [22]. It was suggested that such a feed-forward loop (FFL) [19,20], a well-known local feature of many biological networks, may have a beneficial function. An FLL consisting of a TF and a miR could act as a switch for developmental and other programs in cells, since it may acquire biological systems with robustness to noise by means of canalization of perturbations [22]. We wanted to check whether in any of the significant miR-TF partners discovered above, the miR and its partner TF may regulate each other. We determined how many of the miR–TF partner pairs (out of 104 pairs in the TargetScan dataset and 916 pairs in the PicTar dataset) had a conserved TF binding site of the partner TF in the putative upstream regulatory region of the partner miR (see Materials and Methods for definition of miRs' upstream putative regulatory regions). Interestingly, we found that ten of the TF–miR pairs in the TargetScan dataset (9.6% of the pairs), and 75 out of 916 pairs in the PicTar dataset (8.2%) fulfilled that additional requirement (see Figure 4). To establish whether this rate was significant, we carried out a randomization test (see Materials and Methods) in which we computed, in 10,000 randomized sets of TF–miR pairs, the rate of formation of a regulatory interaction between the TF and the miR. In the TargetScan network, we obtained a modest *p*-value of 0.024; however, in both PicTar networks we obtained the minimal possible *p*-value, <10^−4^, i.e., in all 10,000 randomizations we got a rate of direct regulatory interaction between a TF and the miR, which was lower than the original data (see corresponding z-scores in Figure 4). Thus, the cases in which a TF and a miR co-occur in a highly significant number of target genes was associated more often than random with a direct regulation between the TF and the miR's promoter. We named this feed-forward loop “FFL TF → miR.” The significance of this motif is robust to “noise” in the input, assessed by the method originally used for network motifs in Escherichia coli [20] (see Materials and Methods).

We were also interested in the opposite interaction—i.e., the case in which the miR regulates its partner TF. We named this motif “FFL miR → TF.” We determined how many of the miR–TF partners had a predicted binding site of the partner miR in the 3′ UTR of the partner TF; it occurred five times in the TargetScan network, and 42 and 48 times in the PicTar networks, using two cutoffs on gene regulatory region lengths. This rate was not significant in the TargetScan network (*p*-value = 0.16), yet it was significant in the PicTar networks (*p*-values 0.0038 and <10^−4^). Interestingly, we also found a composite loop network motif, which we termed “FFL miR ← → TF,” in which the pair of partners regulate each other, to be significantly over-represented in the PicTar network; it appeared seven times in the PicTar network (see Figure 4).

In the next step, we looked for another type of network motif, that we termed an “indirect FFL,” in which the TF's regulation on its partner miR is exerted via another mediator TF. We looked to see if any of the miR–TF partners in the network had a conserved TF binding site in a promoter of at least one other TF, which in turn has a conserved binding site in the promoter of the partner miR. Significantly, this architecture was very common in our networks; 30 of the TF–miR partners in the TargetScan network (28%) and 201 partners in the PicTar network (22%) were connected in a regulatory path between the TF and the miR via another TF. We tested the significance of these results by a randomization test, similar to that described above (see Materials and Methods), and received a *p*-value of 1.3 × 10^−3^ for the appearance of the indirect FFL in the TargetScan network, and *p*-value < 10^−4^ for the PicTar network (see Figure 4). For the full list of motifs see Tables S3–S8.

### Expression Analyses Supports miR–TF and miR–miR Predicted Regulatory Interactions

**Figure 5:**
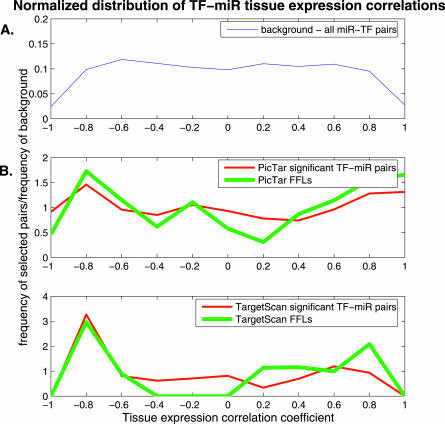
Tissue Expression Correlations between miRs and TFs miR tissue expression in brain, liver, thymus, testes, and placenta were taken from [34]. mRNA tissue expression was taken from [35]. (A) Background distribution of all possible miR–TF pairs for which expression profiles can be derived. (B,C) Normalized histograms of correlation coefficients; the same distribution as in (A) was made, yet only for significantly co-occurring miR-TF pairs (red), and FFLs (green) in the PicTar (B) and TargetScan (C) networks. The figure shows the proportion of the various correlation coefficients divided by the background distribution depicted in (A).

We next analyzed the expression profiles of TF–miR partners. Expression data across human tissues and organs has recently become available for miRs [34] and is also available for protein coding mRNAs [35]. Fortunately, for all the five healthy tissues (brain, liver, thymus, testes, and placenta) for which miRs expression was assayed, mRNAs were measured too. We could thus calculate the correlation coefficient between the expression profiles of each mRNA and each miR, and in particular between all TF–miR partners. For background statistics, we first calculated correlations between all pairs of miRs and TFs in the expression dataset (i.e., not necessarily the TF–miR partners identified above) and obtained their distribution, and found, as may be expected, a distribution that is centered on zero (Figure 5A). On this background we show the distribution of correlation coefficients between expression profiles of TF–miR partner pairs (Figure 5B and 5C). Strikingly, we found that TF–miR partner pairs tended to have high correlation coefficients between them, but, curiously, there was also a tendency for strong negative correlations in some of these pairs. These two tendencies were further enhanced when we inspected only the TF–miR pairs that are connected through an FFL. Given that some TFs can act as activators and others as repressors, and given that miRs may act at the level of translation inhibition or transcript degradation, both negative and positive correlations between TF–miR partners may be mechanistically rationalized.

We further used the same miR tissue expression data to shed light on the co-occurrence and avoidance of miR pairs. We tested whether pairs of miRs that are either highly correlated in their expression levels or anticorrelated to each other across human samples have particularly high co-occurrence or avoidance *p*-values. We found an encouraging correspondence, whereby miR pairs that were positively correlated in expression had a significant tendency for high co-occurrence, whereas miRs with negative correlation in tissue expression typically tended to deliberately avoid residing in shared 3′ UTRs (Figure S5). These observations provide experimental support for miR pairs and TF–miR regulatory interactions that were initially predicted based on sequence information alone.

## Discussion

We provide here a comprehensive characterization of both global and local structural properties of the network of combinatorial regulatory interactions spanned by miRs and TFs. We discovered extensive interactions between miRs and between miRs and TFs, and realize that thousands of human genes are subject to their regulatory effects. Inspection of the distributions of predicted miR sites in human genes' 3′ UTRs revealed hundreds of target hubs [24] in the human genome, genes that appear to be controlled by multiple regulators—miRs in the present case. Curiously, the current target hubs show highly nonrandom representation of specific gene functionalities. Particularly, genes related to development and genes that regulate transcription are enriched among the set of target hubs. These findings constitute another demonstration of the recent concept [24] that suggests that genes that exert extensive regulation on crucial processes are themselves often heavily regulated. So far this has been discussed in the context of the yeast transcription network; this study extends the scope of this concept to the case of miRs in mammalian genomes. In addition, given that each method of target prediction has its own rate of false positives, target hubs, which are predicted to be targeted by multiple miRs, are more likely to actually represent true targets of miR silencing.

The network of extensive regulatory interactions observed here between transcriptional regulators (TFs) and post-transcriptional regulators (miRs), is another interesting global feature. Altogether we estimate that the number of human genes that are under combined regulation at the transcriptional and posttranscriptional silencing levels is between ∼1,000 and ∼4,000 (i.e., ∼12% to ∼43% of the ∼9,000 analyzed genes, according to the TargetScan and PicTar networks, respectively). Overall, ∼9,000 genes were included in the present analyses. These are genes that are currently predicted to have at least one binding site for a known miR. Considering the fact that the collection of mammalian miRs is yet incomplete, and the fact that human specific miRs were not included in the analysis, we anticipate that the true number of human genes that are subject to a dual TF–miR regulation were underestimated in this study. For comparison, we recently estimated that in the Saccharomyces cerevisiae genome about 13% of the genes are subject to regulation at the combined transcriptional and posttranscriptional level [31], albeit with different mechanisms of posttranscriptional regulation operating in this organism, which does not have the miR silencing pathway.

We also examined local properties of the regulatory network, the network motifs. The network motifs described here are different from those originally described [18–20] in that they are composed of a TF and a miR instead of two TFs, as in the original case. We have shown here that network motifs are not only significantly abundant, but also that, according to their current definition, each of them is involved in the regulation of a large set of targets. Interestingly, TF and miR pairs that participate in network motifs show a significant tendency toward high tissue expression correlations or anticorrelations of the two regulators, providing essential experimental support to combinations predicted solely based on sequence information.

Motifs in which the miR regulates its partner TF constitute a type II coherent FFL [18]. In this case it seems that a miR that silences a set of genes posttranscriptionally also silences the transcriptional regulator of these genes, presumably to also prevent de novo transcription of its target genes. This design may be used to minimize leaky transcription of genes in space and time when their expression is undesired. For example, this mechanism could be useful in determining developmental fate in differentiation boundaries as also suggested by [22,23,36].

The motifs in which the TF has a binding site in the promoter of its partner miR corresponds to the incoherent type I FFL (assuming that the TF is a positive regulator). Interestingly, in the S. cerevisiae transcription network this circuit is the second most highly abundant FFL [18]. An intriguing question is what may be the reason for the observed abundance of this circuit in which a TF regulates its partner miR? On the face of it, such regulation appears wasteful if the TF is a positive regulator, since the TF activates an entire set of genes and also a miR that may shut down those target genes. However, if a temporal gap in the activation time of the target genes and the miR exists, then the circuit may be utilized for useful regulatory purposes. For instance, if the TF activates first the target genes and only later the miR (e.g., due to higher affinity, [20]), during a process in which the TF's concentration builds up, the activation of the miR may be timed to obtain a desired delayed shutdown of the regulated genes. We have recently considered similar wiring in the cases of antisense RNAs, another type of regulatory transcripts, and TFs that regulate them in conjunction with their overlapping sense transcripts [37]. The opposite situation, in which the TF positively activates the miR first and only later the target gene, may also be of interest as it can act as a buffer for noisy fluctuations in the levels of the targets; as long as the mRNA level of the target gene is below the inhibition capacity of the miR, fluctuations in its expression levels would not be further propagated. Further, in cases where the miR works predominantly as a translation inhibitor, a controlled mechanism for “just in time” translation for multiple genes is needed for certain functionalities. For example, the miR translation inhibition mechanism was suggested to facilitate localized translation in mammalian dendrites, and to play a crucial role in synaptic plasticity [38]. Such a circuit of coregulating TF–miR in an FFL, where the miR is transcribed by the TF in parallel to the set of mutual targets, could function in featuring localized translation to a whole pathway of regulated genes. Interestingly though, we can point out an example of one indirect FFL we discovered, where a brain-related TF, CREB (CREBATF) [39], partners with a miR that is known to be expressed in the brain, miR-125b [40]. CREBATF was predicted by us to regulate miR-125b through STAT3, which interestingly is also within the list of mutual targets of both miR125b and CREBATF, indicating an even more complex design.

One of the FFLs that came out of our analysis is a composite loop in which the TF regulates the miR and the miR appears to regulate the TF (i.e., a TF ← → miR motif). The circuit consists of the TF E2F and miR-93. miR-93 is part of a cluster of three miRs, miR-106b, miR-93, and miR-25, which lie in close proximity to each other inside an intron of the *MCM7* gene. This network motif was found as an FFL TF → miR in the TargetScan network and as a composite loop in the PicTar network, where all three miRs in the cluster were predicted to target E2F (specifically E2F1 and E2F3). miR-93 cluster members are also homologous to two other genomic miR clusters, one of which is miR cluster 17/92 [41]. Recent evidence suggests a tight regulatory connection of cluster miR-17/92 and E2F [42–45]. E2F1, 2, and 3 were shown to directly upregulate the expression of the miRs encoded in this cluster, while these miRs in turn were shown to act in a feedback loop and to target E2F1–3 mRNAs [42,43]. It was suggested that this feedback may play a role in the major decision mediated by E2F (induction of cellular proliferation or apoptosis). Here we would like to suggest that this intricate regulatory circuit might have another layer to it; in addition to being targeted by the miR-17/92 cluster, E2F family genes might also be targeted by miR-93 cluster members, which share similar seeds. In turn, the miR-93 cluster is transcribed from an intron of the *MCM7* host gene, which is a verified target of the E2F family [46]. Moreover, here the architecture is more complex, as it also includes a set of mutual target genes, through which E2F and the miR-93 cluster may exert their regulatory roles.

Future experimental work will allow the examination of the predictions generated here and the establishment of their precise regulatory roles.

## Materials and Methods

### miRs and their predicted targets.

miRs and their predicted targets were taken from two previously published studies: TargetScan [8,9] (http://www.targetscan.org) and PicTar [7] (http://genome.ucsc.edu). Both resources predict and assign target genes to miRs based on evolutionary conservation between human, mouse, rat and dog. TargetScan targets were downloaded 21 September 2006 and gene symbols were converted to RefSeq IDs using UCSC mysql databases. PicTar targets were downloaded 25 September 2006 from the UCSC hg17 database [7,32] where they are presented as the picTarMiRNA4Way track.

### Target hubs analysis.

Target hubs were defined as genes which are targeted by more miRs than the 99th percentile of the maximal value in 100 randomizations of the columns in the miR to gene assignment matrix; each preserved the total number of targets per miR. According to this procedure, in the TargetScan dataset, target hubs were defined as genes which are targeted by more than 15 miRs (there were 470 such genes), and in the PicTar dataset, target hubs were defined as genes targeted by more than 20 miRs (834 genes). For original and randomized distributions see Figures 1A and S1A.

We wanted to check whether the target hubs contain many miR target sites merely because they have, on average, longer 3′ UTRs. For that purpose, the length of 3′ UTRs for all RefSeq genes was retrieved from UCSC hg17. We performed a randomization test on this 3′ UTR length data, in which we randomly picked a set of genes from the data with distribution of 3′ UTR length that was as similar as possible (see below) to that of the target hubs. For each such set of genes we calculated the average number of different miRs predicted to target them. We repeated this randomization procedure 100 times, and the distribution of average number of miRs was derived (Figure S3). The figure shows that these values are significantly lower than the average of the real target hubs, indicating that the length is neither necessary nor sufficient for a gene to be a target hub.

We generated 100 random sets of genes with length distributions similar to that of the target hubs by the following procedure. For each target hub with UTR length, L_TH_, we defined a set of genes with similar UTR length, which included all the genes in the dataset with a UTR length equal to L_TH_, or longer up to an additional 5% of L_TH_ (genes which did not have such sets were excluded from the analysis). Then, we randomly chose a representative from each set to be included in the randomized version of target hubs. miR density in the 3′ UTRs of genes was calculated as the number of miRs targeting a gene divided by its 3′ UTR length. The 3′ UTR length was extracted from the UCSC database.

When defining high density target hubs we chose the density cutoffs to be the top 85th percentile of the entire distribution of densities. We note that this distribution included only genes that participated in our analyses and thus does not contain genes with a density of zero (i.e., zero predicted sites in the UTR).

### Degree-preserving matrix randomization.

To determine a *p*-value on the co-occurrence rate of a pair of two miRs, we first defined a co-occurrence score. We chose the Meet/Min score [29,30], which is formulated in the main text, and calculated it on the matrix of miR to target genes. For the purpose of *p*-value calculations we defined a null model of randomized matrices, which preserves the matrix statistics such that for each gene the number of miRs targeting it, and for each miR the number of genes it targets remains the same as in the original data. This model was first introduced as a randomization model for networks [20], which preserved all in and out degrees in a given network, and thereby controlling for the possibility that significance of a phenomenon may be merely attributed to the degree distribution in the network. Randomized matrices were created by the edge-swapping procedure, starting from the original matrix of miR to target gene predictions. We randomly picked two pairs of miR and target gene, miR_i1_–gene_j1_ and miR_i2_–gene_j2_, and, after verifying that miR_i1_ does not already target gene_j2_ and miR_i2_ does not already target gene_j1_, we performed the switch of an edge in the matrix, so that after the swap there is a “0” instead of “1” in the positions i_1_,j_1_ and i_2_,j_2_ in the matrix, and a “1” instead of a “0” in the positions i_1_,j_2_ and i_2_,j_1_ in the matrix. To decide how many swapping events were needed before the matrix was “well randomized,” we monitored the number of edges that were actually swapped and compared it with the number of changed edges in a randomly shuffled matrix. We followed this number during the swapping steps and realized that it plateaued at about 100,000 steps. Thus, in all subsequent analyses we repeated the swapping procedure for 100,000 steps.

During the calculation of the Meet/Min score for a pair of miRs in the original data, we excluded genes that contained a match to the two miRs if the two sites physically overlapped on the target's 3′ UTR. In addition, we filtered out from the analysis pairs of miRs whose seeds were identical (overlap of seven out of seven nucleotides, positions 2–8 of the miR). These two precautions were taken to eliminate the possibility of overestimating the significance of the rate of miR co-occurrence due to seed sequence similarity between different miRs.

After having calculated the co-occurrence *p*-values and avoidance *p*-values for all possible miR pairs, we controlled for multiple hypotheses using FDR and only pairs that passed FDR of 0.05 were considered to be significantly co-occurring or avoiding.

### Significant miR–TF co-occurring pairs.

For the task of identifying miR–TF pairs that significantly co-occur in a high number of target genes, a *p*-value was calculated (using a cumulative hypergeometric test) on each pair of regulators as we did before for pairs of TFs [14]. The hypergeometric *p*-value was calculated after the RefSeq genes were mapped to a unique set of Gene IDs, to reduce redundancy in the set. In the miR–TF *p*-value calculations, the total number of genes in the hypergeometric analysis was calculated as the number of genes that appeared (i.e., had at least one binding site) in both datasets. Genes that appeared only in the TF dataset or in the miR dataset were excluded and were not counted. We used FDR to correct for multiple hypotheses testing, and determined the set of significant pairs of coregulators.

We also calculated co-occurrence *p*-values for all possible miR–TF pairs using the new randomization method presented above. Specifically, both the matrix which assigns TFs to genes and the matrix with assignments of miRs to genes were subjected to 100,000 iterations of the edge-swapping procedure. In total we generated 1,000 such pairs of randomized matrices. The co-occurrence *p*-value of a given TF–miR pair is the fraction of the randomized matrix pairs in which this pair's Meet/Min score was higher than the pair's Meet/Min score in the original matrices, and the corresponding z-score is the difference between the original Meet/Min score and the mean of the score in the randomized matrices, divided by their standard deviation.

Most reassuringly, when checking the overlap of these significant pairs with the significant pairs that passed FDR cutoff of 0.3 using the hypergeometric model, we saw that the overlap was very high; it was more than 72% for PicTar and 92% for TargetScan. For subsequent analyses of network motifs (FFLs and indirect FFL search), we chose all the pairs that passed FDR of 0.3 in the hypergeometric test in the three datasets (see Transcription factor binding sites section below), and that passed FDR of 0.3 (*p*-value < 6 × 10^−3^) in the PicTar 10 kb set, and minimal *p*-value (< 10^−3^) in the PicTar 5 kb and TargetScan sets, as these already had an extremely high overlap (>93%) in the hypergeometric derived set.

The final set of significant pairs in the miR–TF network is presented in FDR *q*-value cutoffs of 0.1, 0.2, and 0.3. With *q*-value of 0.1 we obtained 20 TF–miR pairs with significant *p*-value using the TargetScan dataset, and 267 using the PicTar 10 kb dataset, and 70 using the PicTar 5 kb dataset. With a *q*-value of 0.2 we obtained 60 TF–miR pairs with significant *p*-value using the TargetScan dataset, and 555 using the PicTar 10 kb dataset, and 261 using the PicTar 5 kb dataset. With 0.3 we obtained 104 TF–miR pairs with significant *p*-value using the TargetScan dataset, and 916 using the PicTar 10 kb dataset, and 497 using the PicTar 5 kb dataset.

### miRs clusters and regulatory regions.

**Figure 6:**
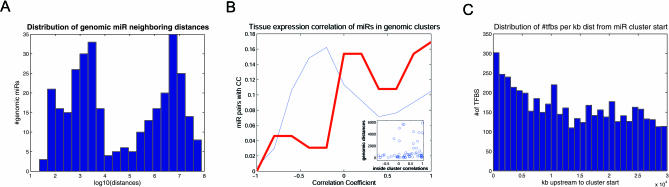
Analysis of miR Clusters in the Human Genome (A) Distribution of distances between all neighboring pre-miR genes in the human genome. (B) Distribution of tissue expression correlations between pairs of miRs: all possible pairs in the data (thin blue line) and pairs of miRs which reside in shared clusters (thick red line). In the inset are shown tissue expression correlations between pairs of miRs in the same genomic clusters versus distances between them. (C) Distribution of number of conserved TFBS 30 kb upstream of the 5′ most nucleotide in each miR clusters. Conserved TFBSs were taken from UCSC hg17.

As was shown in the past [41], miRs may be clustered on the genome, and are often transcribed as one unit. Therefore, to predict regulatory regions of miRs (i.e., proximal as well as potentially more distant promoters or enhancers) we had to first cluster miRs on the human genome. We mapped all 461 pre-miRs in miRBase (http://microrna.sanger.ac.uk, accessed June 2006) [47,48] onto the human genome and clustered them according to physical proximity (genomic locations of miRs were taken from UCSC hg17 and some miRs were mapped from hg18 back to hg17 using the UCSC “lift genome” web service). Two pre-miRs, that are consecutive on the genome, were considered belonging to the same cluster if the distance between them was shorter than a cutoff, provided that they are transcribed from the same strand. We kept adding miRs to clusters until we hit the first distance that was larger than the cutoff. To learn a meaningful cutoff from the data, we plotted the distribution of distances between all neighboring pre-miRs in the genome. Interestingly, we found the distribution to be bimodal—distances below and above 10 kb (on a log scale, Figure 6A) were highly represented in contrast to a lower representation at about 10 kb. This indicated that a reasonable cutoff on the distance between two adjacent miRs that still belong to the same cluster may be 10 kb. Using this clustering procedure we generated 301 clusters, the majority of which (∼82.39%) consists of a single miR; the cluster with the highest number of miRs contains 43 miRs (see Figure S7 for the distribution of number of miRs per cluster). In a previous study, which was based on 207 miRs (compared with the 461 used here), miRs were clustered using a different cutoff [49]. When we repeated our cluster analysis with the current set of miRs, with the previous cutoff, we got similar clustering, 94% of the present clusters are identical to the clusters generated with the alternative cutoff and average cluster lengths are very similar (unpublished data).

Reassuringly, using expression data of miRs across tissues [34] we found that miRs that belong to the same cluster have a significant tendency to be coexpressed compared with miRs that do not map to shared clusters (Figure 6B). This tendency is preserved even in cases where miRs that belong to the same cluster are relatively far from each other on the genome (Figure 6B, inset).

We have then defined, as a putative regulatory region of miRs, the sequence that lies 10 kb upstream of the 5′ most pre-miR in each miR cluster. The 10 kb promoter length was determined from the data as follows. A distribution of number of conserved TFBS upstream of clusters was generated (Figure 6C). We found that the number of conserved TFBS gradually declined as a function of the distance from the putative 5′ end of the cluster, with a plateau obtained at about 10 kb upstream. The distribution was rather noisy, probably due to the fact that primary-miR transcripts are much longer than the precursor miR we relate to (e.g., the primary transcript of the miR-17–92 cluster is C13orf25, which is 6,795 bp long [45]), and thus the transcription start site (TSS) taken here is only crudely defined. We considered the presence of a TFBS in a miR promoter only if such occurrence was conserved in mouse and rat, as taken from the UCSC hg17 conserved track in the relevant regions.

### Transcription factor binding sites.

We used predicted binding sites for all human mouse and rat PSSMs from TRANSFAC [13] version 8.3, as they are defined by the UCSC hg17 genome assembly, in the tfbsConsSites (http://genome.ucsc.edu/) and tfbsConsFactors. All RefSeq genes genomic locations were taken from hg17. To determine the length of upstream regulatory regions, we measured the number of conserved TFBS upstream RefSeq genes as a function of distance from TSS (see Figure S6). The result shows that the signal decays and plateaus between 5 kb and 10 kb upstream of the TSS. We hence chose to work with two alternative cutoffs of promoter length, 5 kb and 10 kb. The regulatory regions thus defined probably consist of proximal promoters as well as distant enhancers. The recent Affymetrix (http://www.affymetrix.com) promoter chip for detection of ChIP experiments with TF binding in human promoters also consists of probes that span 10 kb of regulatory regions, and future experiments with this chip and as many TFs as possible will allow a better delineation of regulatory regions boundaries. Although we used regulatory regions which are longer than the common definition, our use of evolutionary conservation filter gives confidence in the present regulatory region definitions.

### Feed-forward loop statistics.

FFL TF → miR: for all the significant pairs of coregulators (i.e., TF–miR partners that co-occur in a significantly high number of targets) we investigated whether the TF has a binding site in the putative promoter of the miR cluster from which the miR partner is transcribed. In some cases in which the mature miR sequence is transcribed from more than one genomic locus, all possible regulatory regions of the relevant miR clusters were examined. In addition, each PSSM may belong to a family of PSSMs, with similar binding sites, representing the same TF (a family was defined as several PSSMs representing the same TF, as determined from the UCSC hg17 tfbsConsFactors track). Thus, PSSM–miR pairs are treated as TF–miR pair, and given a pair of PSSM–miR partners, we say that the PSSM's TF regulates the miR if at least one of the PSSMs that corresponds to that TF has a match in the regulatory region of the miR partner (the same procedure was carried out in the randomizations described below).

For testing the FFL miR → TF configuration, we had to connect first between TRANSFAC PSSMs and the genes encoding the TFs that bind these PSSMs. For that, PSSMs were mapped to the TF they represent which in turn was mapped to a SwissProt ID. These two mappings were done using the UCSC hg17 tfbsConsFactors track. These SwissProt IDs were then mapped to RefSeq IDs, for which the data on miR targets was maintained. This information served also in the process of indirect FFL search; for each of the TF–miR partners, we checked whether the miR is regulated by another mediator TF, which in turn is regulated by the partner TF. We note that not all TFs had a corresponding SwissProt ID in the UCSC hg17 tfbsConsFactors track, and therefore not all pairs served as candidates for the FFL miR → TF and the indirect FFL; only in 74 of the 104 (71%) TargetScan significant pairs, and in 680 of 916 (74%) of the PicTar pairs, could the PSSM be mapped to a RefSeq gene.

The following procedure was used for the calculation of the significance of the FFLs and indirect FFL in the PicTar and TargetScan miR–TF networks. Since there were 104 and 916 pairs of miR–TF partners in the two respective networks, we have drawn 10,000 times the same number of random pairs of TFs and miRs out of all the possible pairs in each network. The number of each FFL and indirect FFL was recorded in each randomization and a *p*-value (and a corresponding z-score) on the hypothesis that a given network motif is over-represented in the network was taken to be the number of random sets with a greater or equal number of motifs in it.

### miR and mRNA tissue expression data.

The expression profiles of 150 miRs across five healthy human tissues and organs (brain, liver, thymus, testes, and placenta) were previously measured using miR-dedicated microarrays [34]. miRs from the chips were mapped to PicTar and TargetScan; they cover 154 and 87 of the miRs in the two respective datasets. In addition, we used data from [35] for human mRNAs expression across the same set of tissues. Both sets of expression data were column centered (chip-wise centering: each chip's values were divided by the chip mean to account for differences in chip intensities) and then log2 transformed. Regarding mRNA expression chips, we particularly focused on genes coding for the TFs that participated in our analysis. Using the above mapping of PSSMs to their corresponding TF genes, we had a total of 127 TFs that could be matched to at least one probe set in the mRNA expression dataset [35]. We examined the tissue expression correlation of all significantly co-occurring miR and TF pairs for which we had an expression profile. When more than one gene was attributed to the same TF, we chose for each pair of TF and miR the one with the highest absolute value of correlation coefficient out of all options. We did that consistently both for the background statistics of all possible TF–miR pairs and for the predicted TF–miR partners. In total we calculated correlation coefficients for 361 such TF–miR partners out of 916 partners in PicTar, and for 30 out of 104 partners in TargetScan. The miR expression data [34] consisted of five healthy tissues, and HeLa cells, while the mRNA study that we focused on [35] overlapped with the miR data only in the five tissues. Therefore when we compared expression between miRs and TFs we only used the five healthy tissues, and when we compared expression of miR pairs we used all six samples.

### Noise-tolerance analysis.

The assignments of miRs to targets are known to be of limited accuracy [21] . We thus wanted to assess the noise tolerance of our results. We adopted a procedure previously utilized for the case of network motifs in the bacterial transcription network [20]. We experimented with different percentages of the connections in the network that were randomly removed or added and the significance of the present FFL motifs was assessed for each case. Similarly to the findings in the E. coli network, we found that up to 20%–30% of the edges can be added or removed without appreciable effect on the FFL significance.

## Supporting Information

Dataset S1 Pajek Input File for the miR Co-Occurrence Network, the TargetScan Dataset (Significant Co-Occurring miR Pairs with FDR *q*-Value 0.05) All networks in the Dataset files can be interactively viewed using the Pajek software, which can be freely downloaded from (http://vlado.fmf.uni-lj.si/pub/networks/pajek/).
(12 KB TXT)

Dataset S2 Pajek Input File for the miR Co-Occurrence Network, the PicTar Dataset (Significant Co-Occurring miR Pairs in FDR *q*-Value 0.05) (20 KB TXT)

Dataset S3 Pajek Input File for the Network of miR–TF Coregulating Pairs This graph depicts all the significant miR–TF pairs in the TargetScan network, in addition to all the FFLs. A red node is a TF and a green node is a miR, and a blue edge is drawn if the TF and the miR are co-occurring partners. A yellow edge connects between a TF and a miR if, in addition to having a high rate of co-occurrence, they also form a FFL TF → miR; a pink edge represents the FFL miR → TF motif, while orange edge represents a FFL miR ← → TF (in all cases the set of target genes is not explicitly shown).
(16 KB TXT)

Dataset S4 Pajek Input File for the Network of miR–TF Coregulating Pairs This graph depicts the 100 most significant pairs in the PicTar (10 kb) network, in addition to all the FFLs.
(86 KB TXT)

Dataset S5 Pajek Input File for the Network of miR–TF Coregulating Pairs This graph depicts the 100 most significant pairs in the PicTar (5 kb) network, in addition to all the FFLs.
(55 KB TXT)

Figure S1 Distribution of miRs to Target Gene Assignments in the PicTar Dataset (A) Distribution of the number of different miRs regulating each target gene in the PicTar dataset. The thick red line represents the distribution in the original datasets, while each of the thin blue lines represents the distribution in one of the column-randomized matrices. The matrix contains only genes with at least one predicted site in their 3′ UTR. In each randomization, we shuffle the assignment of miRs to their targets, keeping constant the number of targets per miR.
(B) Distribution of number of targets per miR in the PicTar dataset. In the thick red line we depicted the original distribution, while each blue thin line represents the distribution in one of the 100 row-randomized matrices, which preserve the distribution of number of miRs targeting each gene.
(1.6 MB EPS)

Figure S2 miR Binding Sites and 3′ UTR Length in the TargetScan and PicTar Datasets A dot plot depicting number of miRs targeting each gene and its 3′ UTR length of the target hubs, high miR number target hubs in green, high density target hubs in red, genes that are target hubs according to both criteria in magenta and the rest of the genes in blue for the (A) TargetScan dataset and (B) PicTar Dataset.
(C) Distribution of the miR densities in the 3′ UTRs of target hubs (thick red line) and all the genes (thin blue line) in the PicTar dataset (all genes included in this figures have at least one miR site predicted in their 3′ UTR). The log10 densities were binned into bins of 0.1, and relative frequencies were plotted.
(1.6 MB EPS)

Figure S3 Click here for additional data file. miR Binding Sites in Target Hub Genes in the TargetScan and PicTar Datasets
Mean number of miRs targeting each of the genes that are target hubs (red bar), in the entire set of analyzed genes (green), and a distribution of that mean in random gene sets with the same (or very similar, see Materials and Methods) distribution of 3′ UTR lengths as the target hubs (blue) in (A) the TargetScan dataset and (B) the PicTar dataset. For elaborated procedure see Materials and Methods.
(1.6 MB EPS)

Figure S4 miR Pairs Interaction Network in the PicTar Dataset (A) The miR pairs interaction network in the PicTar database.
(B) Degree distribution in the PicTar miR combinatorial regulation network (co-occurring miR pairs that passed FDR of 0.05)
(1.6 MB EPS)

Figure S5 Positively Correlated miR Pairs Tend To Have Significant Co-Occurrence *p*-Values while Negatively Correlated Pairs Tend to Avoid Residing in the Same 3′ UTRs Highly expression correlated miR pairs tend to have significant co-occurrence or *p*-values, while negatively correlated pairs tend to have significant avoidance *p*-values. The figures depict the Kolmogorov-Smirnov *p*-values for the hypotheses that correlated miR pairs have lower co-occurrence *p*-values than the rest of the pairs. Correlated pairs were defined according to correlation cutoffs (depicted on the *x*-axis), with positively correlated pairs in blue, negatively correlated pairs in green. Positively correlated miR pairs tend to have significant co-occurrence *p*-values in both TargetScan (A) and PicTar (C). Negatively correlated pairs tend to have significant avoidance *p*-values in both TargetScan (B) and PicTar (D).
(3.9 MB EPS)

Figure S6 Distribution of Number of Conserved TFBS 30 kb Upstream of TSS of RefSeq Protein-Coding Genes (1l KB EPS).

Figure S7 Distribution of Number of miRs per Cluster As seen, ∼82% of the 301 clusters contain a single miR.
(12 KB EPS)

Table S1 Significant Co-Occurring miR Pairs in the TargetScan Dataset (30 KB XLS)

Table S2 Significant Co-Occurring miR Pairs in the PicTar Dataset (38 KB XLS)

Table S3 Significant Co-Occurring miR–TF Pairs in the TargetScan Network (32 KB XLS)

Table S4 Significant Co-Occurring miR–TF Pairs in the PicTar Network, Taking 10 kb Regulatory Regions for Protein Coding Genes (172 KB XLS)

Table S5 Significant Co-Occurring miR–TF Pairs in the PicTar Network, Taking 5 kb Regulatory Regions for Protein Coding Genes (103 KB XLS)

Table S6 Indirect FFLs in the TargetScan Dataset (22 KB XLS)

Table S7 Indirect FFLs in the PicTar Dataset Taking 10 kb Regulatory Regions for Protein Coding Genes (47 KB XLS)

Table S8 Indirect FFLs in the PicTar Dataset Taking 5 kb Regulatory Regions for Protein Coding Genes (29 KB XLS)

## Abbreviations

FDR: false discovery rate
FFL: feed-forward loop
miR: microRNA
PSSM: position specific scoring matrices
TF: transcription factor
TFBS: TF binding sites
TSS: transcription start site
UTR: untranslated region
GO: Gene Ontology
